# Stabilizing decontamination foam using surface-modified silica nanoparticles containing chemical reagent: foam stability, structures, and dispersion properties†

**DOI:** 10.1039/d0ra07644a

**Published:** 2021-01-06

**Authors:** In-Ho Yoon, Suk Bon Yoon, Youngho Sihn, Man-Soo Choi, Chong-Hun Jung, Wang-Kyu Choi

**Affiliations:** Decommissioning Technology Research Division, Korea Atomic Energy Research Institute 111, Daedeok-daero 989 Beon-gil, Yuseong-gu Daejeon 305-353 Republic of Korea ihyoon@kaeri.re.kr +82-42-868-8281; Decommissioning Research Institute Preparation Unit, Korea Hydro & Nuclear Power Co., Ltd. (KHNP) Gyeongju 38120 Republic of Korea

## Abstract

The stabilization of decontamination foams containing a chemical reagent is a crucial requirement for their use in the decontamination of nuclear power plants. We have investigated the effects on decontamination foam stability of adding silica nanoparticles (NPs) modified with various functional groups, namely propyl (–CH_3_), amine (–NH_2_), and thiol (–SH) groups. The surface properties of these silica NPs were characterized with ATR-FTIR, solid NMR, and TGA analyses. We also established that the agglomeration in such foams of the amine-modified silica NPs is weaker than that of the other modified silica NPs due to their thorough dispersion in the liquid film. Further, the foam containing amine-modified silica NPs was found to be stable for 60 min at a pH of 2, *i.e.* under decontamination conditions. The bubble structure analysis showed that this decontamination foam has a bubble count that is approximately 5–8 times higher than the foams containing NPs modified with the other functional groups, which indicates that the decontamination foam with amine-modified silica NPs has the best foam structure of the three investigated foams. The well-dispersed and smaller amine-modified silica NPs enhance the foam stability by providing a barrier between the gas bubbles and delaying their coalescence. In contrast, the thiol- and propyl-modified silica NPs form aggregates with large diameters that reduce the maximum capillary pressure of coalescence and hence decrease the foam stability.

## Abstract 2

The stabilization of decontamination foams containing a chemical reagent is a crucial requirement for their use in the decontamination of nuclear power plants.

## Graphical abstract

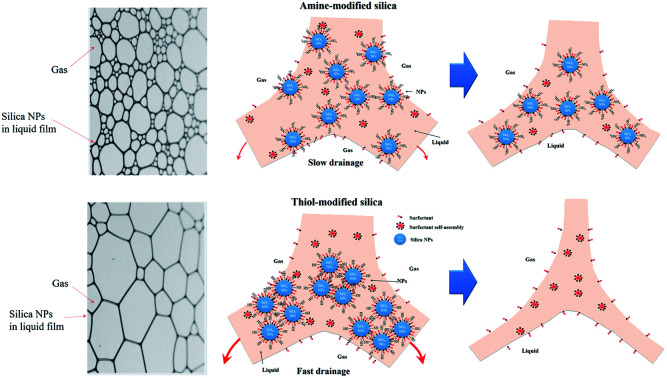

## Introduction

The surfaces of the equipment and buildings of nuclear power plants or facilities are contaminated by radioactive species and must be decontaminated with chemical reagents during decommissioning, which usually generates large amounts of liquid waste. Decontamination with a foam consisting of 10% (v/v) liquid and 90% (v/v) gas can be used as an alternative method to reduce the amount of liquid waste. The contact time between the chemical reagents in the foam and the contaminated surfaces must be long because the decontamination efficiency of foams is relatively low due to their low concentrations of chemical reagents.^1^ Numerous strategies for improving the stability of decontamination foams have been reported, particularly the addition of stabilizers such as gelatin,^2–4^ alginate,^5,6^ xanthan gum,^1^ flocculated suspensions,^7^ LAPONITE®,^8–10^ and silica NPs.^11–14^ It is difficult to treat secondary wastes containing organic matter after foam decontamination, so inorganic stabilizers are preferred over organic stabilizers.

In our approach, the foam consists of a two-phase system of surfactants and silica NPs; the NPs act as surface modifiers that increase the stability of dispersions in the two-phase system.^15–18^ However, because the role of NP–surfactant interactions in the interfacial behavior of fluid/fluid systems is not yet well understood, the mechanism of NP–surfactant interactions is the subject of increasing research attention.^19^ The complexity of such interfacial behavior in two-phase surfactant–NP systems is increased by the electrostatic attraction and repulsion of ionic surfactants. In general, unmodified silica NPs, such as hydrophilic silica, are not surface active, but they can strongly influence the interfacial behavior in two-phase surfactant–NP systems.^20,21^

Although a number of valuable studies of the interfacial behavior of two-phase surfactant–NP systems have been conducted, their focus has mainly been negatively charged silica NPs and the cationic surfactant cetyl trimethyl ammonium bromide (CTAB).^20,22–28^ According to the results of these studies, the electrostatic attraction between the negatively charged surfaces of the silica NPs and the cationic surfactant (CTAB) enhances surfactant adsorption on the surfaces of the silica NPs. In contrast, anionic surfactants do not interact with the surfaces of negatively charged silica NPs. The interfacial tension of a sodium dodecyl sulfate (SDS) solution below its critical micelle concentration is lower in the presence of negatively charged silica NPs.^29–33^

**Fig. 1:**
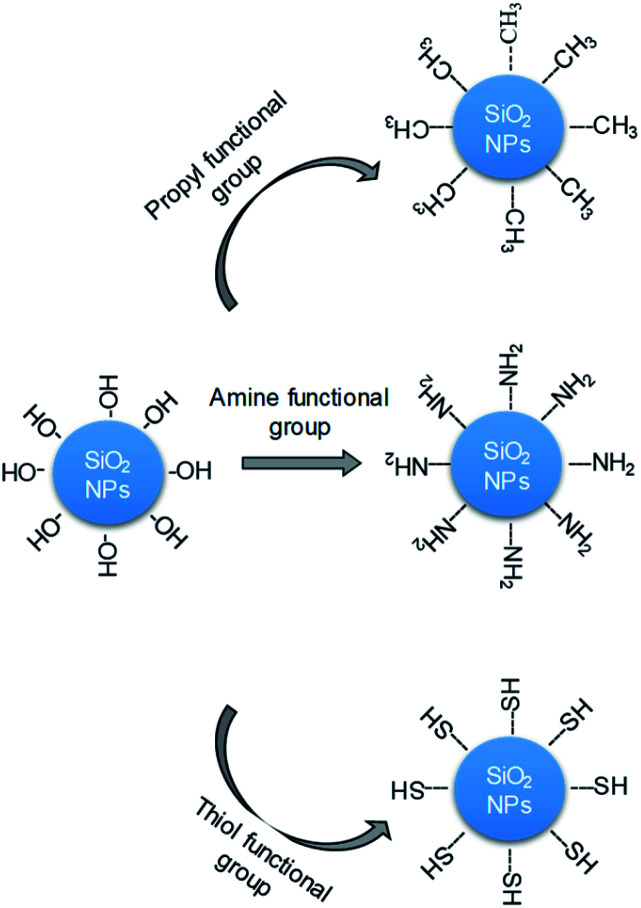
Schematic diagram of the modification of the surfaces of silica NPs (bare) with propyl (–CH_3_), amine (–NH_2_), or thiol (–SH) groups.

The change in the hydrophobic and hydrophilic properties that result from surfactant adsorption is the main reason that silica NPs adsorb onto the liquid interface.^25,34^ In this study, the surface-modified silica NPs act as surface-modifying agents that enhance the interfacial properties of the two-phase surfactant–NP system and thereby increase its stability.^22,23,25,26,35^ Various surface treatments can be used to adjust the dispersion of the silica NPs and thus the foam stability, particularly the addition of various functional groups that promote electrostatic interactions with their surfaces.^36–41^ Recently, researchers reported the enhancement of foam stability that results from modifying silica NPs with positively charged polyethylenimine^36^ or enhancing their partial hydrophobicity with the addition of (CH_3_)_2_SiCl_2_.^40^ However, surface-modified silica NPs have not previously been used as stabilizers in decontamination foams. In this study, we synthesized silica NPs with surfaces that had been modified with various functional groups with the aim of increasing their dispersion in decontamination foams by minimizing the interaction between the silica NPs and the surfactant, and thus enhancing the foam stability. We analyzed the physical properties of the silica NPs, and examined the resulting foam stability and dispersion behavior with the aim of developing a new decontamination foam formulation (Fig. 1). We investigated (i) the characteristics of the surface-modified silica NPs, (ii) the surface charges, size distributions, and dispersion properties of these silica NPs in decontamination foam solutions, and (iii) the foam stability and structures of the decontamination foams containing modified silica NPs. This is the first time surface-modified silica NPs have been used to enhance the stability of decontamination foams. The new formulation with amine-modified silica NPs was found to exhibit enhanced foam stability because of the increase in the dispersibility of the silica NPs.

## Experimental

### Synthesis and modification of silica nanoparticles

Non-porous silica NPs were synthesized by using an established method.^42^ In a typical procedure, silica seed NPs were synthesized by adding tetraethylorthosilicate (TEOS; 25 mL) into a mixture consisting of absolute ethanol (1000 mL), deionized H_2_O (10 mL), and 28 wt% NH_4_OH (25 mL), and then stirring for 24 h at the ambient temperature. The silica seed solution (24 mL) was then suspended in a solution of absolute ethanol (1000 mL), DI H_2_O (80 mL), and 28 wt% NH_4_OH (40 mL). After stirring for 0.5 h, tetraethylorthosilicate (40 mL) was added into the above reaction mixture and stirring was continued for 6 h under the same conditions. Finally, tetraethylorthosilicate (80 mL) was added to the mixture, which was then stirred for 6 h. Various functional groups were added to the silica NPs by reacting the particle surfaces with the silane coupling agents (3-aminopropyl)trimethoxysilane (+96%, 281778, Aldrich) for amine functionalization (–NH_2_), (3-mercaptopropyl)trimethoxysilane (+95%, 175617, Aldrich) for thiol functionalization (–SH), and *n*-propyltriethoxysilane (97%, 539317, Aldrich) for propyl functionalization (–CH_3_). In these surface modifications, 1.0 g synthesized silica NPs in 100 mL DI water was magnetically stirred with 1% mol_silane_ mol_silica_^−1^ silane coupling agents. The concentrations of the reacted silane agents were typical of silica surface modifications. The modified silica NPs were separated by centrifugation at 100 000*g* for 15 min. The samples were washed with ethanol/DI water and freeze-dried over 24 h.

### Preparation of the decontamination foams

The surfactant Elotant™ Milcoside 100 (EM 100) (alkyl polyglucoside) for the preparation of the decontamination foam was provided by LG Household & Health Care, and it contained 10 alkyl chains on average. All solutions were prepared by using deionized (DI) water from a Milli-Q water system. To examine the effects of the silica NPs modified with amine, thiol, and propyl groups on foam stability, 100 mL of DI H_2_O was added to a 1.0% v/v solution of EM100 at pH 2 adjusted with 1 M HNO_3_ in a 100 mL beaker.

### Foam stability and structure analyses

In all the foam stability and structure experiments, the foam and its liquid height/volume were analyzed by using a Dynamic Foam Analyzer (DFA-100, KRŰSS, Germany), while the foam structure was simultaneously characterized (particularly the bubble size distribution and the thickness of the liquid layers within the foam). Air was injected through a sintered glass filter at the bottom of a cylindrical glass vessel (40 mm inner diameter) during foaming. The diameter and thickness of the glass filter were 4.4 cm and 0.4 cm respectively. The pore size of the filter was 16–40 μm. The initial liquid volume was 0.06 L; the gas flow was 0.2 L min^−1^ during foaming and was stopped after 60 s. The foam height and liquid height were analyzed by using a light-emitting diode panel and a photon detector positioned at the front and back of the column, and were continuously checked by analyzing the light transmission through the glass column.^43,44^ The total foam height was 20 cm (approximately 0.25 L) in all determinations.

A camera with a scanning area of 1.05 cm × 0.75 cm was positioned 5.5 cm above the glass filter to determine the foam size distribution. By making use of the principle of total reflection, a prism enables the 2D structure analysis of foams located along the path of the light. The glass and liquid have similar refractive indices, so the light incident on a foam lamella is partially diffracted and thus transmitted into the bubble. In contrast, glass and air have different refractive indexes, so the light hitting the gas bubbles is completely reflected. The subsequent high-contrast images were investigated by using foam analysis software, and the bubble size distribution was recorded every 2 s.^44^

### Analysis

The synthesized silica NPs and those modified with amine, thiol, and propyl groups were characterized by using various analytical tools. Scanning electron microscopy (SEM) (S-4300, Hitachi, Japan) was used to determine the shapes and sizes of the NPs. The surface areas of the NPs were determined by using the Brunauer–Emmett–Teller (BET) method with a Micromeritics ASAP 2420 (USA) analyzer at 77 K. Pre-heated vacuum degassing was performed on the samples at 423 K for 2 h prior to the isotherm measurements. The transmittance spectra of the modified silica NPs were recorded with ATR-FTIR (Alpha-P, Bruker, USA). These measurements were carried out at a resolution of 4 cm^−1^ with 64 scans over the range 600–4000 cm^−1^. The extent of functionalization of the silica NPs was investigated with elemental analysis (EA, Flash 2000 Thermo, USA) and thermogravimetric analysis (TGA, TG 209 F1 Libra Netzsch, Germany). In the TGA, the modified NPs were heated from 25 to 800 °C at a heating rate of 20°C min^−1^ in a N_2_ atmosphere. The heating temperatures were maintained at 150, 350, and 550 °C for 30 min to precisely quantify the surface bound components. We calculated the surface densities of nitrogen (–NH_2_), carbon (–CH_3_), and sulfur (–SH), by using the equationSurface density = element mass per functionalized silica mass × molecular weight of element × Avogadro^'^s number ÷ surface area of the silica

The surface area of functional groups was determined to be 15.4 m^2^ g^−1^ by performing BET analyses.

Solid-state proton nuclear magnetic resonance (1H NMR) spectra were recorded with a Bruker AVANCE III HD (Bruker, Germany) at the KBSI Western Seoul Center. The analysis was performed at 9.4 T by using a HX CPMAS probe with a 4 mm o.d. zirconia rotor. Tetramethylsilane (TMS) solution was used as the external reference. The sizes and surface charges of the modified NPs were determined by using a DLS device (Malvern Zetasizer nano-ZS90, Malvern, UK).

The dispersion stabilities of the pure silica NPs and the surface-modified silica NPs were analyzed by using a Turbiscan (Turbiscan Lab, Formulaction, France). Sedimentation phenomena were assessed by using an optical analyzer, Turbiscan Lab (Formulaction, France), which uses multiple light scattering to characterize dispersion conditions. The Turbiscan Lab was used to detect and characterize the destabilization of dispersions with various concentrations, *i.e.* their coalescence, flocculation, creaming, and sedimentation. The viscosity properties of the various foam formulations were analyzed by using a rheometer (Brookfield Eng. & Lab. Inc., LVDV-III ULTRA) at a shear rate of 60/s^−1^ for 2 min (120 points) and Rheocalc 32 software.

## Results and discussion

### Characteristics of the surface-modified silica NPs

**Fig. 2:**
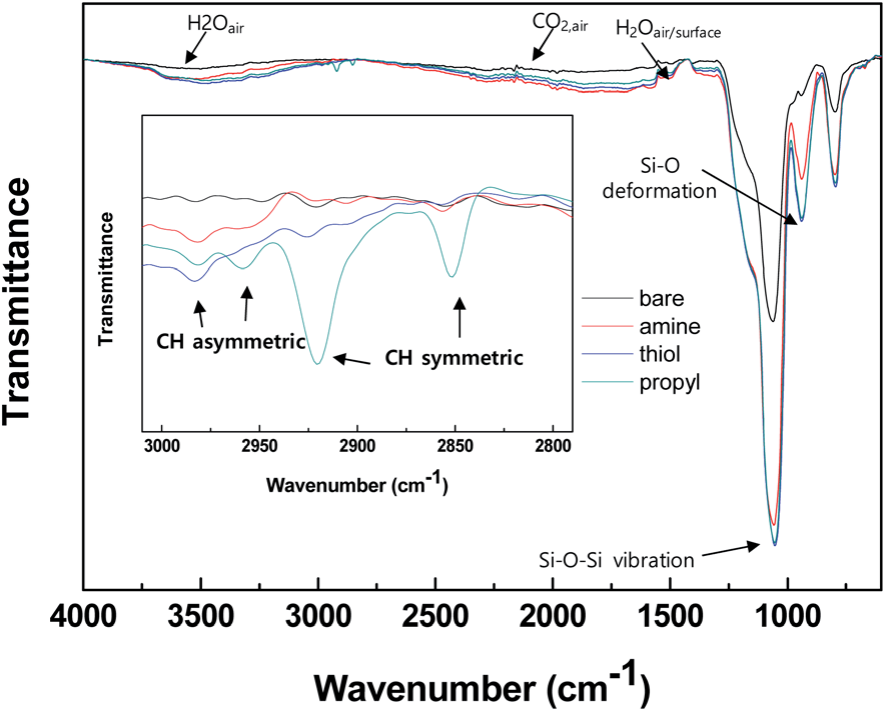
ATR-FTIR spectra of the silica NPs: bare and functionalized with amine, thiol, or propyl groups.

We determined the properties of the bare and modified silica NPs by using various analytical tools, and our results clearly show that the desired functional groups were grafted onto the silica surfaces. Attenuated total reflectance Fourier transform infrared (ATR-FTIR) and solid NMR analyses were used to characterize the functional groups grafted onto the surfaces of the silica NPs. Fig. 2 shows the ATR-FTIR spectra of the bare and modified surfaces of the silica NPs. Intense silica peaks are evident for all samples in the range 800–1100 cm^−1^, particularly the stretching vibrations of Si–O–Si and Si–OH at 1050 and 800 cm^−1^ respectively. Vibration peaks due to the alkyl chains CH_3_ and CH_2_ were observed only for the modified silica NPs in the range 2800–3000 cm^−1^, whereas there are no such peaks for the bare silica NPs (Fig. 2 (inset)). Since the silane coupling agents contain both functional groups and alkyl chain groups, the observed alkyl peaks demonstrate that the silane coupling agents are immobilized on the surfaces of the silica NPs. Bands due to functional groups are not evident in the spectra for the modified silica NPs probably because of their overlap with the intense silica and CO_2_/H_2_O_(air)_ peaks.

**Fig. 3:**
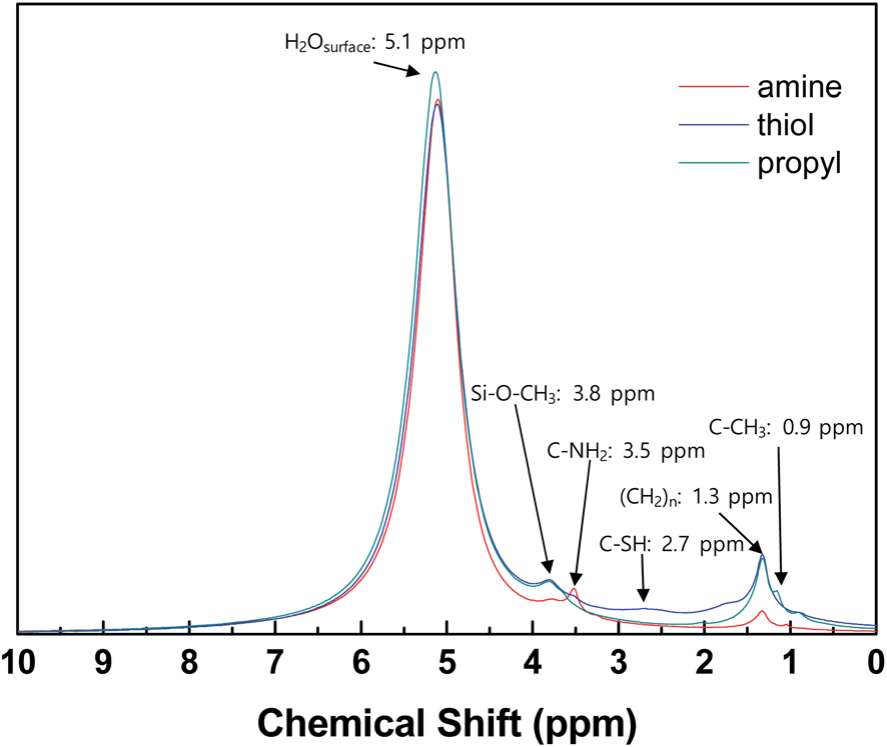
1H NMR spectra of silica NPs functionalized with amine, thiol, or propyl groups.

^1^H NMR analysis was carried out to confirm the presence of grafted functional groups on the surfaces of the silica NPs (Fig. 3). For all the modified silica NP samples, intense signals are evident due to water adsorbed onto the silica NPs and the alkoxy and alkyl chain groups of the grafted silane coupling agents.^45–47^ The spectra of the samples modified with different functional groups contain weak signals at different positions according to the characteristics of the functional groups, which match previously reported values, *i.e.*, 3.5 ppm (–NH_2_ silica), 2.7 ppm (–SH silica), and 0.9 ppm (–CH_3_ silica).^48–51^ These results confirm that the silane coupling agents have been successfully grafted onto the surfaces of the silica NPs.

### Elemental and TGA analyses for the silica NPs modified with various functional groups

**Fig. 4:**
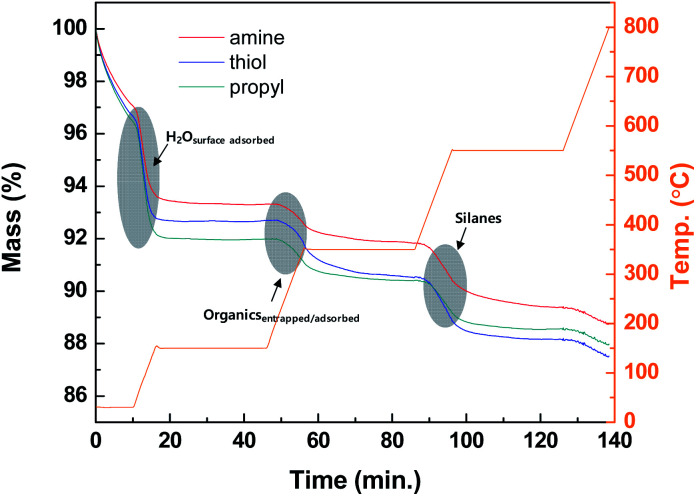
TGA curves for the silica NPs functionalized with amine, thiol, or propyl groups.

The thermolysis profiles shown in Fig. 4 of the modified silica NPs were obtained with TGA to determine the amounts of organic groups on the modified silica NP surfaces. The heating temperatures were maintained at 150, 350, and 550 °C to perform the complete thermolysis of each component of the modified silica NPs. The profiles contain three abrupt mass loss regions. The first mass loss (4%) occurs during the period 0–10 min from 25 to 150 °C and is due to the loss of surface-adsorbed water. The next region (approximately 2%) occurs from 150 to 350 °C and is probably due to the loss of physically entrapped or adsorbed organics such as ethanol or external impurities. The last region occurs from 350 to 550 °C and is ascribed to the loss of the silanes grafted onto the silica NP surfaces. The complete decomposition of the silanes occurs above 450 °C due to the cleavage of the bonds between silicon atoms and hydrocarbon chains.^52^ Thus substantial mass loss (1.81–2.49%) clearly arises for the modified silica NPs in the range 350–550 °C, which confirms that the silanes containing the functional groups were successfully attached to the silica NP surfaces.

Table 1 summarizes the carbon (C), nitrogen (N), and sulfur (S) contents of the bare and modified silica NPs. None of C, N, or S were detected in the bare silica NPs. However, in the modified silica NPs, these elements are present in the added functional groups. The carbon contents for the amine-, thiol-, and propyl-modified silica NPs are 1.27 ± 0.06, 1.59 ± 0.02, and 0.92 ± 0.02% (w/w), respectively. The amine-modified silica NPs contain only 0.24 ± 0.03% (w/w) of N. The thiol-modified silica NPs contain only 0.85 ± 0.06% (w/w) of S. These results demonstrate that the modified silica NPs contain the desired functional groups. As used equation of the surface density, the surface density of NH- (*ρ*_NH_ = 6.7 molecule-S per nm^2^), SH- (*ρ*_SH_ = 10.4 molecule-S per nm^2^) and CH- (*ρ*_CH_ = 10.0 molecule-C per nm^2^) modified silica were obtained following the identical procedure. These surface densities were calculated by using the monolayer value for C_3_–silane grafted silica (2.1–4.2 molecule per nm^2^).^53^ Thus the functional groups are present in multi-layers on the silica NP surfaces. The properties of the bare and modified silica NPs were investigated with various analytical tools and these results clearly demonstrate that the desired functional groups have been grafted onto the silica NP surfaces.

**Table 1:** Elemental analysis results for the bare and modified silica NPs

Silica type	C (%)	N (%)	S (%)
Bare	0	0	0
Amine (–NH_2_)	1.27 ± 0.06	0.24 ± 0.03	0
Thiol (–SH)	1.59 ± 0.02	0	0.85 ± 0.06
Propyl (–CH_3_)	0.92 ± 0.12	0	0

### Surface charges and size distributions of the silica NPs in the decontamination foam solutions

**Fig. 5:**
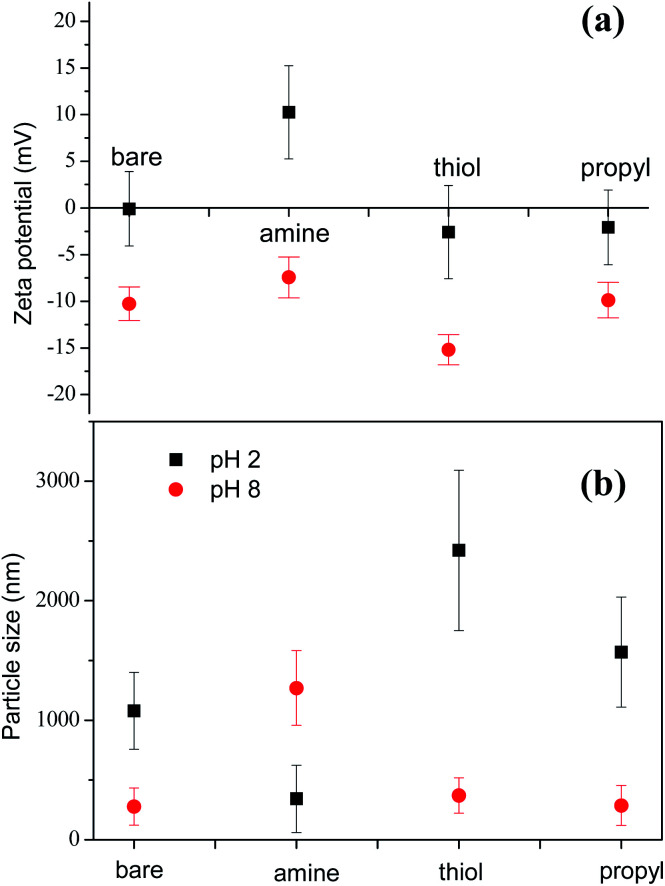
(a) Zeta-potentials and (b) particle size analyses for 1 wt% amine-, thiol-, and propyl-modified silica NPs in 1% v/v EM 100 at pH 2. Values are the averages of the results of three experiments.

Our SEM images show that the sizes of the silica NPs modified with various functional groups are similar and in the range 200–300 nm (ESI, Fig. S1†). However, the results obtained with dynamic light scattering (DLS) for the particle sizes in liquid are significantly different. Before foaming, the surface charges and size distributions of the silica NPs in the decontamination solution were measured by using DLS. Considering their similar surface areas, these dramatically different dispersion behaviors are probably mainly due to the differences between the properties of the surfaces of the modified silica NPs. We determined the zeta-potentials of the silica NPs to analyze their surface charges and to evaluate the effects of pH on their surfaces. At pH 2.0, the amine-modified NPs have a zeta potential of +10.3 ± 5.2 eV, which is higher than that of the bare silica NPs (−0.1 ± 4.0 eV), the thiol-modified NPs (−2.6 ± 4.8 eV), and the propyl-modified NPs (−2.1 ± 3.8 eV) (Fig. 5(a)). These results show that the electrostatic repulsion between the amine-modified NPs is increased by the colloidally positive (+) zeta potentials of the silica NPs, which promotes the dispersion of the silica NPs in the bulk.

Fig. 5(b) shows the sizes of the bare and amine-, thiol-, and propyl-modified silica NPs at pH 2 and pH 8. Particles larger than 1000 nm indicate the formation of big aggregates. Particle size of amine-modified silica nanoparticles at pH 2 was 343.2 nm ± 281 nm. Particle sizes of stable bare, thiol-, and propyl-modified silica nanoparticles at pH 8 were 277.9 nm ± 156 nm, 370.3 nm ± 148 nm, and 287.4 nm ± 167 nm, respectively, comparable to the size of the amine-modified silica nanoparticles at pH 2. Aggregated particle size of silica NPs depends various factors such as primary particle size, temperature, solvent permittivity, particle density, pH, zeta potential, *etc.* Extended Derjaguin–Landau–Verwey–Overbeek (DLVO) theory partly can explain the stability of the particles.^54^ In DLVO theory, total interaction potential is composed of two opposing interaction potentials: attractive van der Waals and repulsive electrostatic interaction. At pH 8, all particles except for the amine-modified silica particles are negatively charged, and they did not form large aggregated microparticles. Zeta potential of the amine-modified silica particles at pH 8 (>−10 mV) is not enough to stabilize the particles, and it showed big aggregated particles.^55^ On the other hand, at pH 2, all particles except for the amine-modified silica particles were not stable due to the small zeta potentials. In case of the positively-charged amine-modified silica at pH 2, it did not aggregate, and the particle size is the smallest.^56^

### Turbiscan analyses of the dispersion of the silica NPs in the decontamination foam solutions

**Fig. 6:**
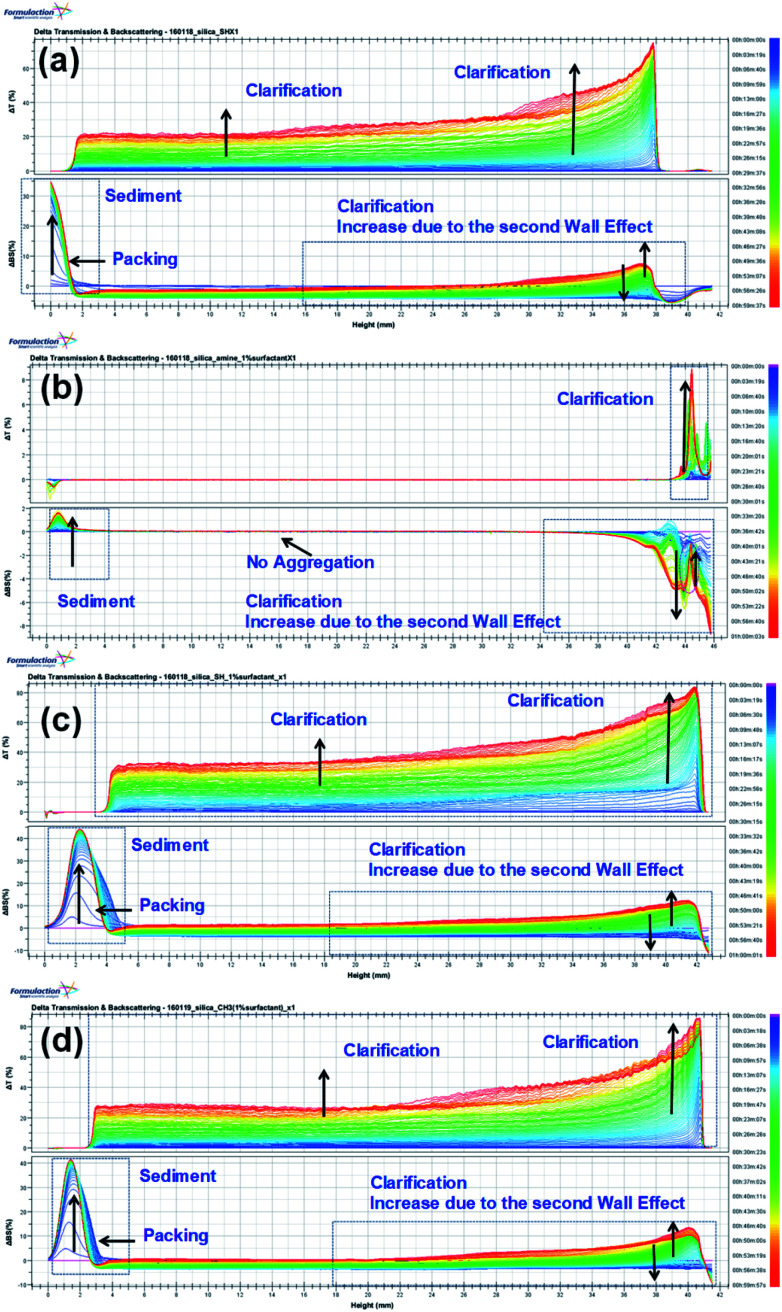
Turbiscan stability kinetics and indexes for the decontamination foams containing 1 wt% silica NPs: (a) bare, (b) amine-modified, (c) thiol-modified, and (d) propyl-modified.

We examined the sedimentation in the foam solutions by using an optical analyzer, the Turbiscan Tower (Formulaction, France), which characterizes the various dispersion states with multiple light scattering. The Turbiscan Tower can be used to detect and characterize destabilization processes (coalescence, flocculation, creaming and sedimentation) in dispersions with various concentrations.^57^ The changes in the transmission (T) and backscattering (BS) light intensity of the foams with 1% amine-, thiol-, or propyl-modified NPs are shown in Fig. 6. The colors in the spectra correspond to different elapsed times. In Fig. 6(a)–(d), the backscattering level decreases at the top (right part of the graph) due to a reduction in the concentration of functionalized silica NPs, so clarification is occurring, whereas it increases at the bottom due to an increase in the concentration of functionalized silica NPs upon sediment formation.

**Fig. 7:**
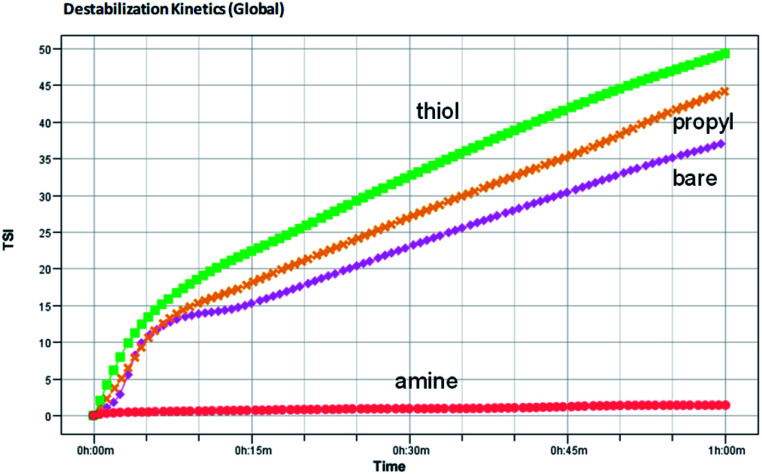
Turbiscan stability kinetics and indexes for the decontamination foams containing 1 wt% bare, amine-, thiol-, or propyl-modified silica NPs.

The global BS decreases due to flocculation and increases due to the wall effect. The change in the global BS for amine-modified silica NPs is smaller than that for the thiol- and propyl-modified silica NPs, which indicates that the amine-modified silica NPs are more stable than the other silica NPs. BS also varies due to sedimentation. The BS at the top of the sample decreases at first but then increases due to the second wall effect after a few minutes. As a result, T also increases at the same sample height. The BS in the middle steadily increases due to the second wall effect, but flocculation was not evident during the analysis. When we ranked the stability of the NPs using TSI (global), the amine-modified silica NPs were found to be more stable than the thiol- and propyl-modified silica NPs. Using the TSI values shown in Fig. 7, we can list the foams in the order of decreasing stability as follows: amine-modified > bare > propyl-modified > thiol-modified. The TSI values of the amine-modified silica NPs in sedimentation are smaller than those of the other silica NPs, which suggests that the sedimentation of the amine-modified silica NPs is slower, so they are better dispersed in complex fluids than the other silica NPs.

### Stabilities of the decontamination foams containing modified silica NPs

**Fig. 8:**
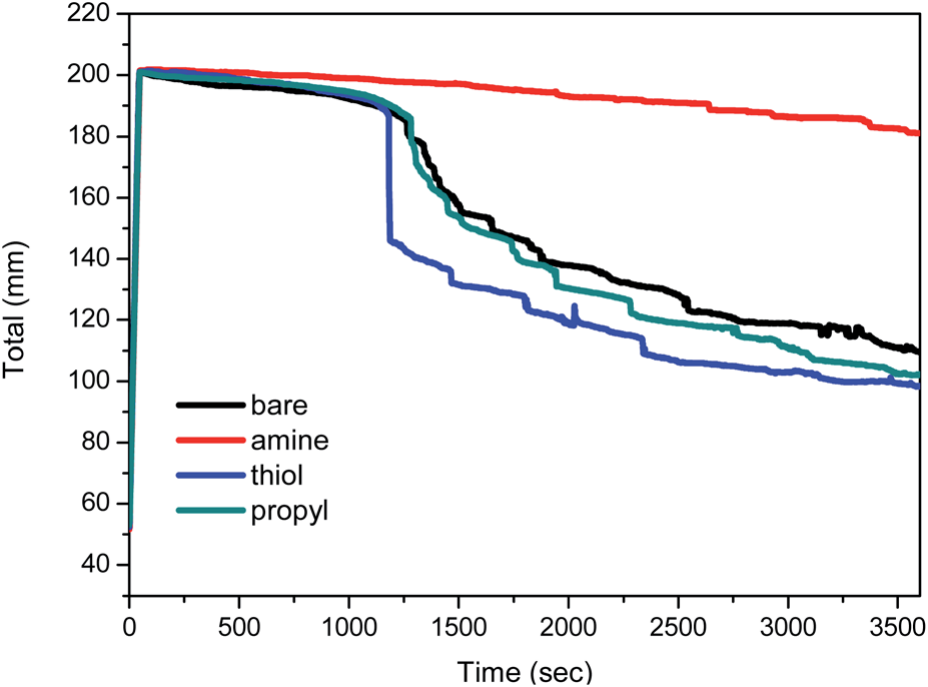
Stability results for foams containing 1 wt% bare, amine-, thiol-, and propyl-modified silica NPs and 1% v/v non-ionic surfactant with a pH of 2. Values are the averages of the results of three experiments.

The variations with time in the total height of the foams are shown in Fig. 8. The total height of the foam containing amine-modified silica NPs is stable for a relatively long period, whereas the heights of the foams containing bare, thiol-, or propyl-modified silica NPs decrease to 50% after 60 min, *i.e.* their foam volumes decreased to 100 mL. In particular, the foam containing 1 wt% amine-modified silica NPs at pH 2 retains its initial height for 60 min. A previous study reported that the presence of a non-ionic surfactant does not affect foam stability at pH 2, 3, or 5, so it can be used under acidic pH conditions.^12^ Ethoxylated fatty alcohols are important non-ionic surfactants and are thought to be hydrolytically stable in the pH range 3–11.^58^ However, low pH values (pH < 3) affect the foam stability when diluted nitric acid is used as the chemical reagent. After the addition of silica NPs, the foam was stable for 1 h. The silica NPs form a dense aggregation in the liquid film at the foam surface; this phenomenon is an important factor that increases the foam stability while preventing foam coalescence.

### Structures of the decontamination foams containing modified silica NPs

**Fig. 9:**
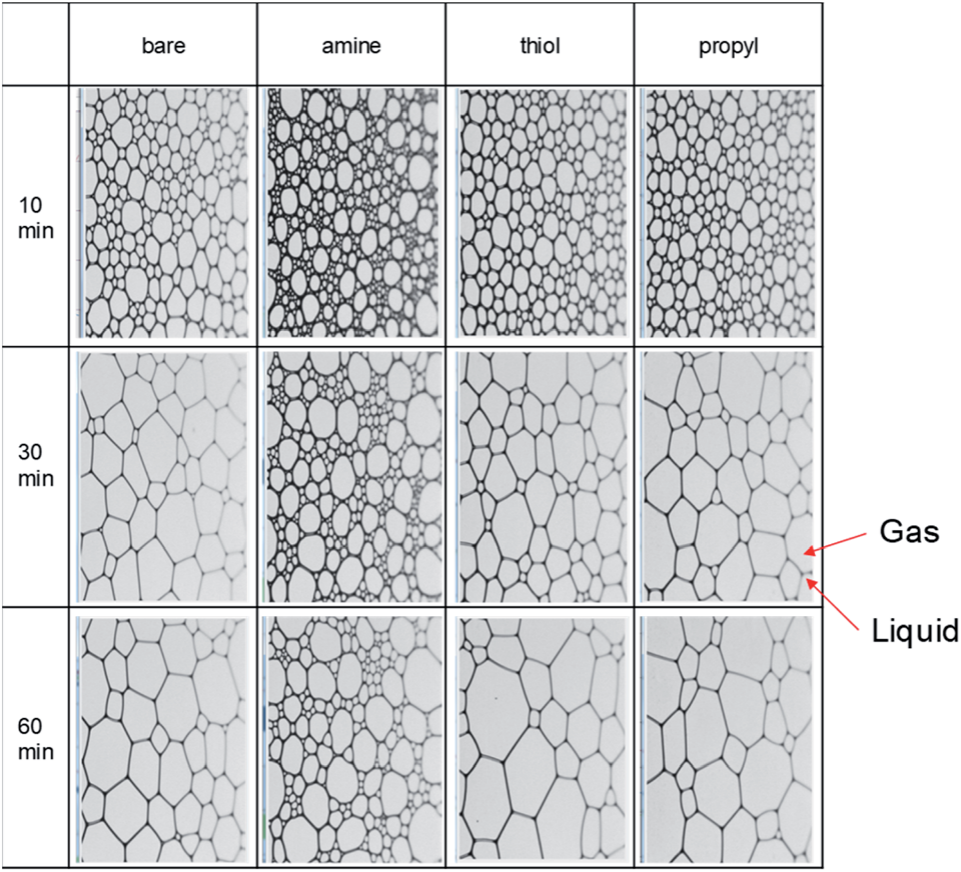
Images of the bubbles and structures in 1% v/v EM 100 (pH 2) containing 1 wt% amine-, thiol-, and propyl-modified silica NPs after 10, 30, and 60 min.

**Fig. 10:**
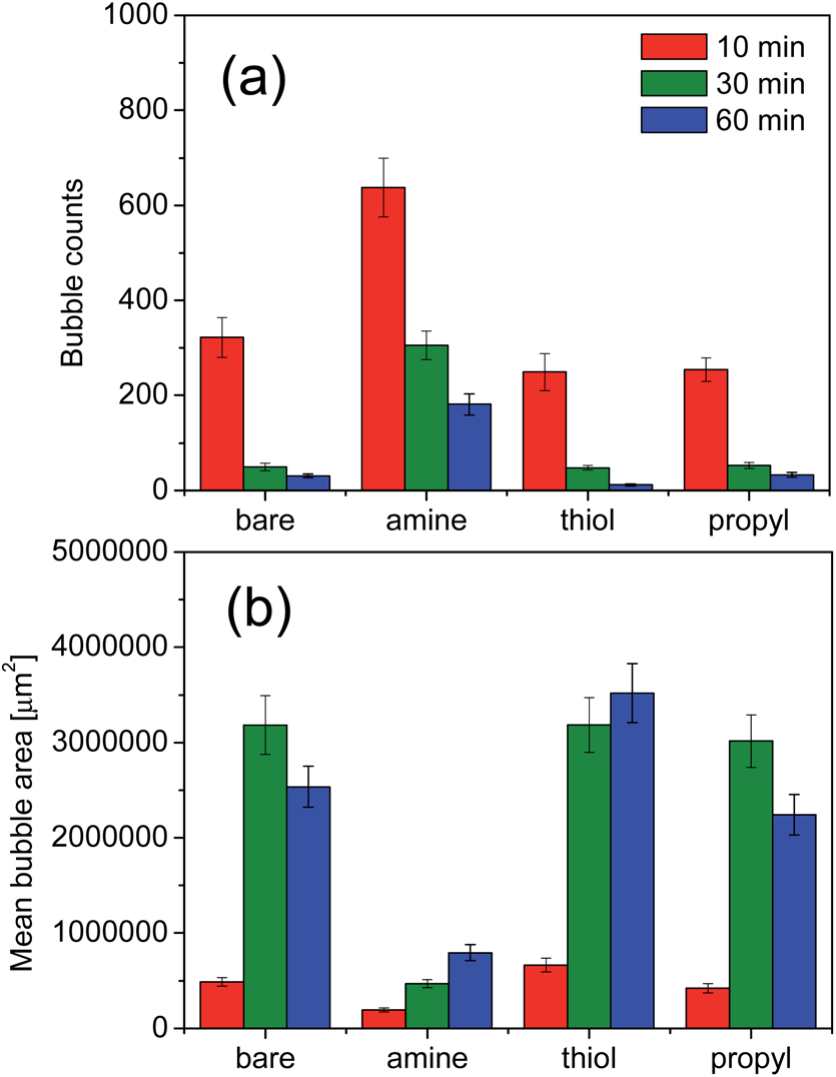
Results of the foam structure measurements at pH 2: (a) bubble counts and (b) bubble areas. Values are the averages of the results of three experiments.

We evaluated the stabilities of the decontamination foams containing surface-modified silica NPs; Fig. 9 and 10 show the variations in the structures and stabilities of the decontamination foams prepared using 1 wt% mol_silane_ mol_silica_^−1^ bare silica NPs and silica NPs modified with amine, thiol, and propyl groups at pH 2 over a 60 min period. Fig. 9 shows the structures of the decontamination foams at times of 10, 30, and 60 min after foaming. Fig. 10 shows the bubble counts and mean areas of the decontamination foams in Fig. 9. Since the efficiency of the removal of contaminants obtained with a decontamination foam is very important, we performed foam stability tests for the decontamination foams prepared in this study under acidic conditions at pH 2. The bubble count for the foam with amine-modified silica NPs, *i.e.*, 200, is approx. 4.4–10 times larger than those obtained for the foams with bare silica NPs (40), thiol-modified silica NPs (20), and propyl-modified silica NPs (45) after 60 min (Fig. 10(a)). This result confirms that the stability of the decontamination foam containing amine-modified silica NPs is better than those of the other formulations we tested. The mean bubble size of the foam with amine-modified silica NPs, *i.e.*, 795 088 μm^2^, after 60 min is approx. 2.5–4 times smaller than those of the foams with bare silica NPs (2 536 079 μm^2^), thiol-modified silica NPs (3 519 523 μm^2^), and propyl-modified silica NPs (2 243 129 μm^2^) (Fig. 10(b)). These results show that the bubble size of the decontamination foam containing amine-modified silica NPs is less than those of the foams containing NPs modified with other functional groups, and that this foam has a higher bubble count.

The structural properties of the bubbles, namely their sizes, numbers and average areas, were determined with a DFA 100. Previous studies have reported that in the absence of silica NPs the bubbles grow and became irregular over time, and that larger bubbles merge with adjacent small bubbles due to pressure differences according to the Young–Laplace theory. In contrast, the presence of partially hydrophobic or surfactant–silica NPs keeps the bubbles small; the added NPs are adsorbed onto the foam surface and improve the deformation resistance of the foam film, thus increasing foam stability.^59^ In addition, the small amount of floc resulting from combining the surfactant with the NPs improves the foam stability by retarding foam coalescence.^60^ The resultant large number of small bubbles with uniform size results in comparatively good foamability and foam stability.^44,61^ For foams containing amine-modified silica NPs, the average count and mean area of bubbles are the highest and lowest respectively of the foams prepared in this study for all analyzed durations. This result indicates that amine-modified silica NPs provide more stability than thiol- or propyl-modified silica NPs and bare silica NPs.

### Mechanism of the stabilization of foams by surface-modified silica NPs

Various mechanisms have been proposed to explain the enhancement of foam stability by NPs. The clusters of NPs form a network inside the liquid film in the foam, which enhances foam stability by preventing coalescence and coarsening.^10^ Dickson proposed that the dominant mechanism of foam stabilization by NPs is the aggregation of NPs at the solution–gas interface, which acts as a barrier preventing film breakage.^62^ Another proposal suggests that the improvement in foam stability in the presence of NPs is due to the decrease in interfacial tension.^23^ Some studies concluded that the partial flocculation of NPs with appropriate size in the bulk and at the interface is the key mechanism in the improvement of foam stability.^10,63,64^ AIYousef reported that the addition of more solid particles or surfactant has a negative impact on foam stability and that an intermediate concentration of NPs with respect to that of the surfactant is required to enhance foam stability.^60^

**Fig. 11:**
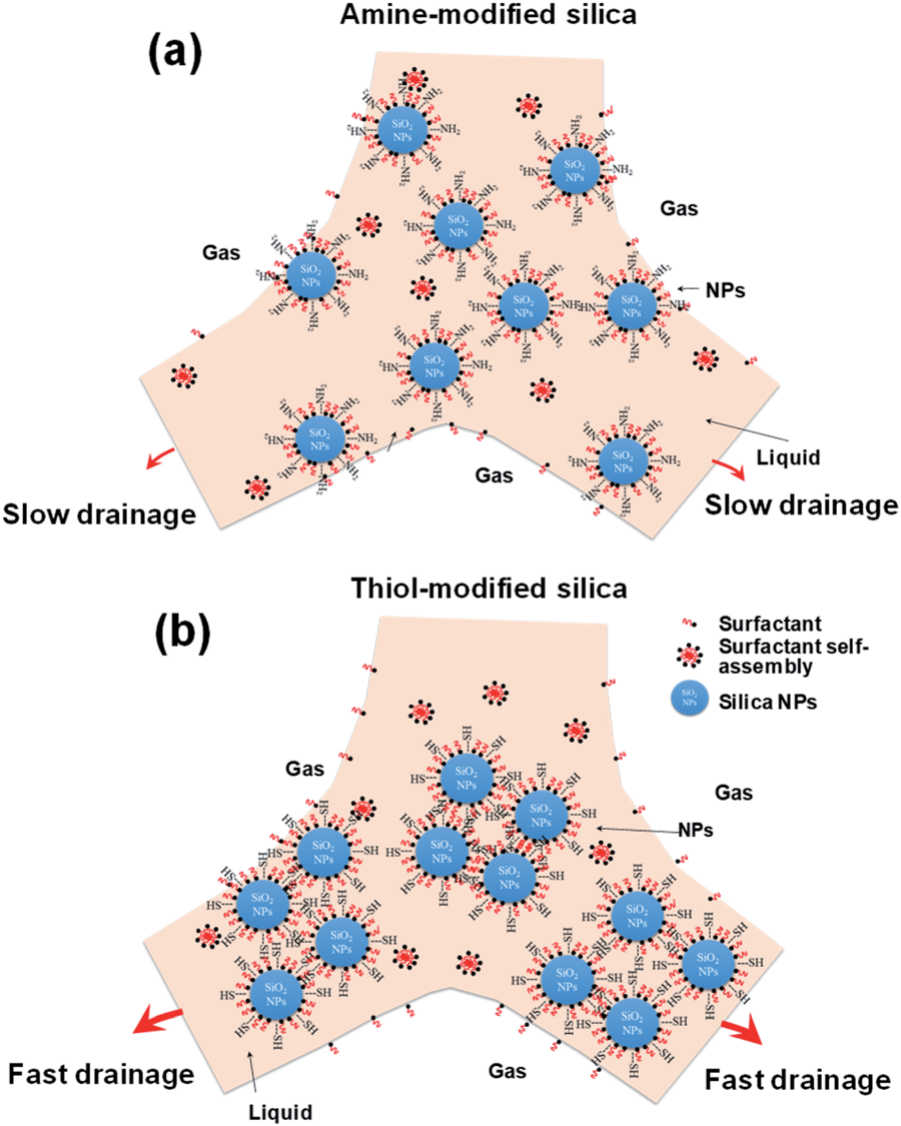
Schematic diagram of the mechanism of foam stability for two different surface-modified silica NPs.

Fig. 11 shows schematic diagrams of the behaviors of highly dispersed silica NPs (amine-modified) and agglomerated silica NPs (thiol- and propyl-modified) in the liquid films of decontamination foams. The results in Fig. 5–7 show that the agglomeration of the amine-modified silica NPs is less than that of the other modified silica NPs due to their thorough dispersion in the liquid film. Thus, well-dispersed and smaller silica NPs persist for a longer time in the liquid film than larger silica NPs, which prevents drainage and coalescence, and thus enhances foam stability. In addition, the viscosity of amine silica NPs increased, because more smaller NPs are present per unit volume (ESI, Fig. S2†). AIYousef showed that the generation of flocs as a result of mixing NPs and surfactant can enhance the foam stability by providing a barrier between the gas bubbles and thus delaying their coalescence. In addition, NPs increase the solution viscosity of thin aqueous films and thereby slow their drainage rate.^60^ As a result, an intermediate concentration of NPs with respect to that of the surfactant enhances the foam stability.

However, the agglomeration of the thiol- and propyl-modified silica NPs occurs rapidly due to their neutral and partially hydrophobic surfaces. Agglomerated particles (1000–2500 nm) undergo rapid drainage and do not remain in the liquid film. AIYousef reported that the aggregation of NPs at these concentrations results in the formation of flocs with large diameters and a reduction in the maximum capillary pressure of coalescence, and so expedites film breakage.^60^ Therefore, even though the viscosity might increase because of agglomeration, the maximum capillary pressure of coalescence will decrease as the radius of the particles increases.

## Conclusions

This study assessed the effects of the addition of modified silica NPs to decontamination foams on their stability with the aim of decreasing the amounts of waste generated by chemical decontamination and increasing the decontamination efficiency. We concluded that the use of amine-modified silica NPs with a non-ionic surfactant in decontamination foams with a pH of 2 provides better foam stability than the addition of bare, propyl-, and thiol-modified silica NPs. The stability of a decontamination foam containing amine-modified silica NPs is approximately 5–8 times higher than foams containing silica NPs modified with other functional groups because of its thorough dispersion and higher viscosity. The addition of amine-modified silica NPs results in a larger number of particles per unit volume and in smaller particles than the other modified silica NPs because they have a partial positive charge, which prevents aggregation between particles and increases their dispersibility in the liquid film of the decontamination foam. Such a decontamination foam with surface-modified silica NPs can be applied to radionuclide-contaminated areas as a highly effective decontamination agent for the reduction of secondary waste.

## Conflicts of interest

There are no conflicts to declare.

## Supplementary Material

RA-011-D0RA07644A-s001
