# Supplementary material for: Stabilizing decontamination foam using surface-modified silica nanoparticles containing chemical reagent: foam stability, structures, and dispersion properties
Source: RSC Adv. 2021 Jan 6;11(3):1841–9. doi: 10.1039/d0ra07644a (PMC8693613; doi:10.1039/d0ra07644a)
Supplement: RA-011-D0RA07644A-s001 [file RA-011-D0RA07644A-s001.pdf]

Electronic Supplementary Material (ESI) for RSC Advances.
This journal is © The Royal Society of Chemistry 2020

Supplementary materials

Figure S1

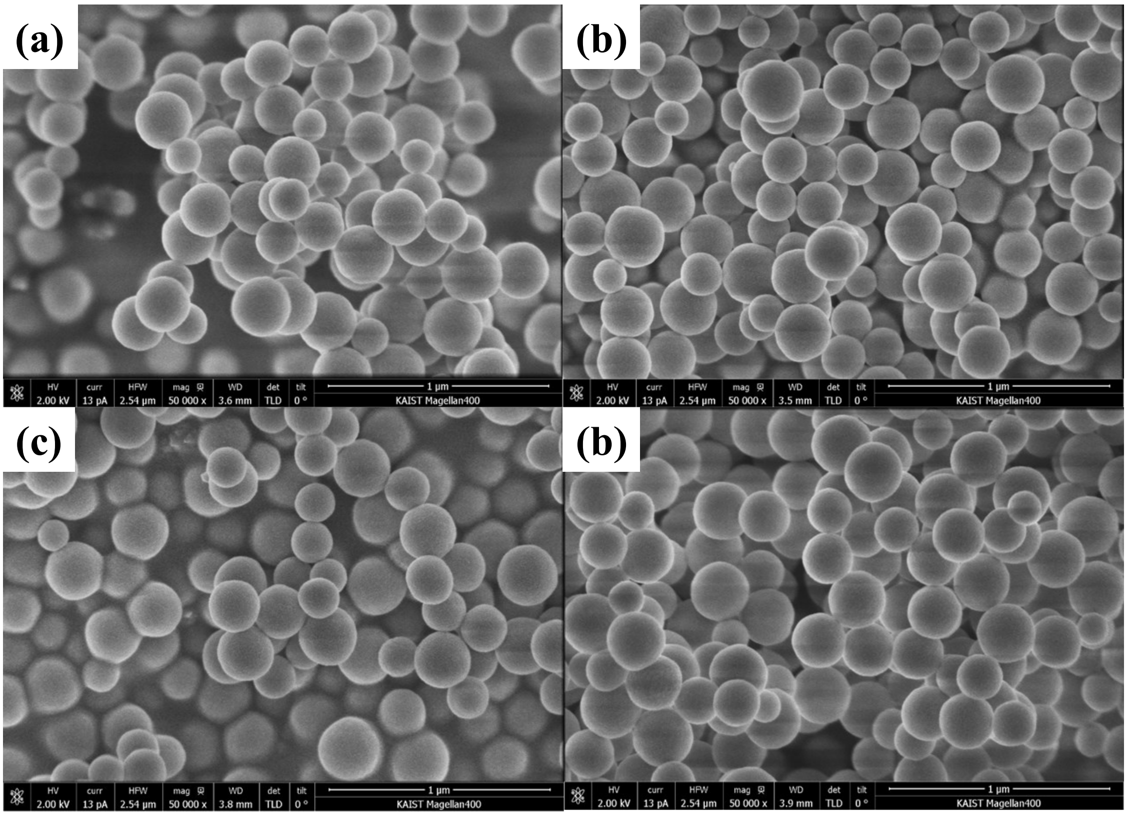

Figure S1. SEM results of the (a) bare, (b) amine, (c) methyl, and (d) propyl modified silica NPs.

Further tests were performed to evaluate the viscosity of the decontamination foam solution with bare and modified silica NPs. The viscosity of the decontamination foam solution with amine-modified silica NPs was greater than that of the bare and other modified silica NPs (Fig. S2), because more silica NPs were existing per unit volume due to the smaller particles. According to these results, the amine-modified silica NPs in the same solid portion increased the viscosity, improving the coalescence resistance. Recently, previous study described that the silica NP diameter has a definite effect on the comparative viscosity of suspensions based on low viscosity liquids. In other words, the smallest particles have the highest viscosity.[1,2] We found the same trend in our results.

Figure S2

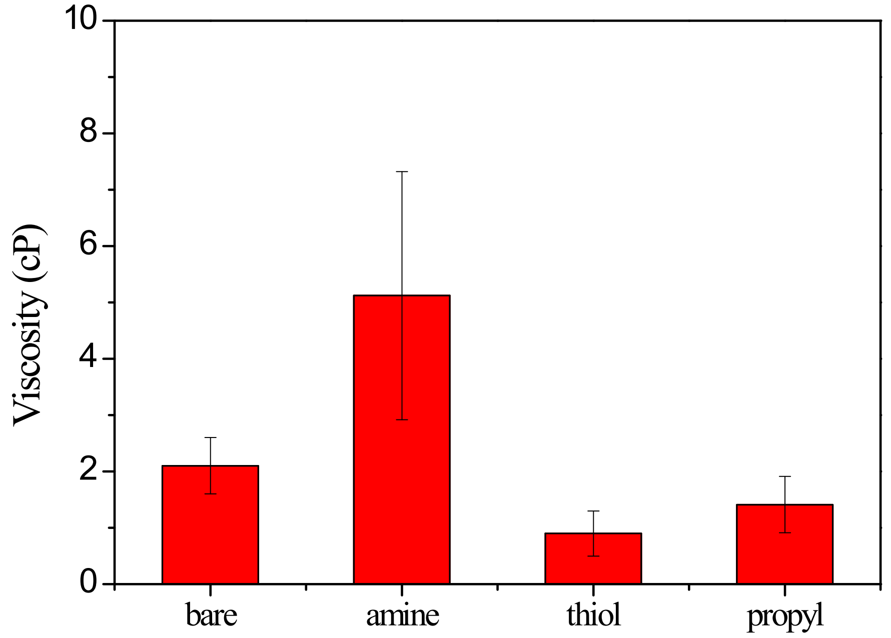

Figure S2. Viscosity of decontamination foam solution with 1 wt.% bare, amine, thiol, and propyl modified silica NPs in 1%(v/v) EM100. Values are the average of triplicate experiment.

References

1. B. J. Konijn, O. B. J. Sanderink and N. P. Kruyt, Powder Technol., 2014, 266, 61-69.
2. P. K. Senapati, B. K. Mishra and A. Parida, Powder Technol., 2010, 197, 1-8.
